# Naringin targets Zeb1 to suppress osteosarcoma cell proliferation and metastasis

**DOI:** 10.18632/aging.101710

**Published:** 2018-12-22

**Authors:** He Ming, Qiu Chuang, Wang Jiashi, Li Bin, Wang Guangbin, Ji Xianglu

**Affiliations:** 1 Department of Orthopedic Surgery, Shengjing Hospital of China Medical University, Heping District, Shenyang 110004, People’s Republic of China

**Keywords:** naringin, osteosarcoma, Zeb1, proliferation, metastasis

## Abstract

Naringin, a citrus bioflavonoid, has anti-inflammatory actions and cardio- and neuroprotective effects. In addition, naringin exhibits multiple antitumor actions in several cancer types, including osteosarcoma, the most common type of bone cancer. Here, we show that naringin inhibits proliferation and invasion and induces apoptosis in human osteosarcoma cells by inhibiting zinc finger E-box binding homeobox 1 (Zeb1), a transcriptional repressor of epithelial differentiation involved in tumor metastasis. Our expression analyses confirm that Zeb1 is highly expressed in osteosarcoma specimens and cell lines. The effects of naringin, which included downregulation of Cyclin D1, MMP2, and bcl-2, where reproduced by siRNA-mediated Zeb1 silencing, whereas Zeb1 overexpression increased proliferation, migration, and Cyclin D1, MMP2, and bcl-2 levels. In addition, naringin administration reduced tumor nodule formation and attenuated the expression of the above proteins in the livers of mice injected with MG63 osteosarcoma cells. Our study provides preclinical evidence for the potential therapeutic application of naringin in the treatment of osteosarcoma.

## Introduction

Although adjuvant chemotherapy has improved osteosarcoma survival rate in recent years, development of multidrug resistance severely impacts prognosis and restricts success of curative attempts [1–3]. Therefore, new and effective drugs to treat osteosarcoma are clearly needed.

Naringin, a bioflavonoid abundant in grapefruit and other citrus, has multiple biological activities. It possesses sedative, antifungal, antispasmodic, and analgesic properties, and provides cardioprotective, neuroprotective, and anticancer effects [4]. In addition, naringin has been demonstrated to inhibit inflammatory responses, prevent bone degeneration, and exert anabolic effects on bone cells [5,6]. Naringin promotes the expression of β-catenin and increase Ser552 phosphorylation on β-catenin in UMR-106 osteosarcoma cells. This led to activation of lymphoid enhancer factor (LEF)/T-cell factor (TCF) transcription factors to stimulate bone development [7]. Naringin abrogates osteoclastogenesis and bone resorption by inhibiting RANKL-induced NF-κB and ERK activation [8], and demonstrated therapeutic potential to attenuate polymethylmethacrylate-induced osteoclastogenesis and osteolysis. There is substantial evidence supporting a role for naringin as an anticancer agent. Studies also indicated that naringin could reduce the release of inflammatory factors and inhibit the growth of W256 carcinosarcoma in rats [4,9,10]. Moreover, growth arrest and apoptosis were common effects of naringin in several *in vitro* and *in vivo* studies conducted on breast, cervical, ovarian, bladder, hepatic, skin, colorectal, and gastric cancer cells [11,12].

Zeb1 (zinc finger E-box binding homeobox 1) is a transcription factor that represses epithelial differentiation and promotes a mesenchymal phenotype [13]. Zeb1 is upregulated in several cancers, where it influences cell motility, cell cycle, and survival, and is an important contributor to tumor invasion and metastasis [14,15].

Studies have shown that Zeb1 can override the G1 checkpoint directly, by stimulating Cyclin D1 expression, and indirectly, by regulating the Wnt signaling pathway [16,17]. Zeb1 was shown to promote the progression of lung cancer by increasing the expression of MMP2, a member of the matrix metalloproteinases family that play an important role in cell migration and facilitate invasion and metastasis of tumor cells [18,19]. Zeb1 has also been shown to be upregulated in osteosarcoma, and to contribute to its development [20,21].

Using human osteosarcoma cell lines as experimental model, in the present study we provide *in vitro* and *in vivo* evidence that naringin suppresses proliferation and metastasis of osteosarcoma cells by inhibiting the expression of Zeb1. Our findings highlight the potential of naringin, a safe and natural flavonoid, for osteosarcoma therapy.

## RESULTS

### Naringin inhibits the expression of Zeb1 in osteosarcoma cells

**Figure 1:**
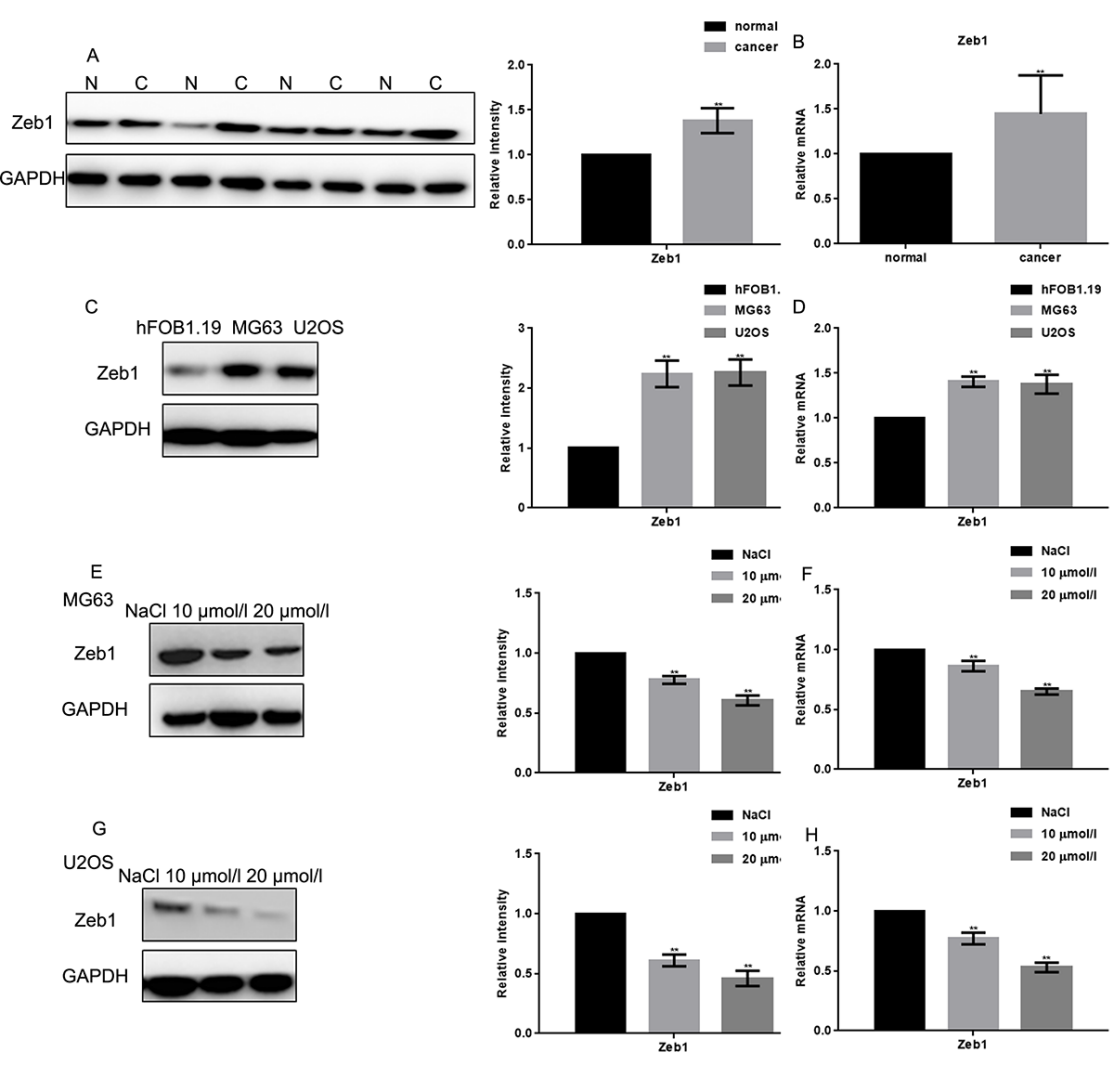
**Naringin inhibits the expression of Zeb1 in osteosarcoma cells.** (**A, B**) Zeb1 expression in 30 human osteosarcoma specimens and their adjacent normal tissue counterparts was detected by Western blot and real-time PCR. **P < 0.05, vs normal tissues. (**C, D**) Zeb1 expression in MG63, U2OS and hFOB1.19 cells, detected by Western blot and real-time PCR. **P < 0.05, vs hFOB1.19 cells. (**E-H**) Zeb1 expression detected by Western blot and real-time PCR in MG63 and U2OS cells treated with NaCl or indicated concentrations of naringin for 24 h. **P < 0.05, compared with NaCl.

The expression of Zeb1 in human osteosarcoma samples was assessed by Western blot and real-time PCR (Figs. 1A, B). Both assays showed that Zeb1 was overexpressed in most samples, although heterogeneity was evident. In cultured cells, both Western blot and real-time PCR showed much stronger Zeb1 expression in osteosarcoma MG63 and U2OS cells than in control hFOB1.19 osteoblasts (Figs. 1C, D). Upon exposure to naringin (10 or 20 μmol/L) for 24 h, Zeb1 protein and mRNA levels were dramatically decreased, in dose-dependent manner, in both osteosarcoma cell lines (Figs. 1 E-H).

### Naringin inhibits proliferation and induces apoptosis in osteosarcoma cells

**Figure 2:**
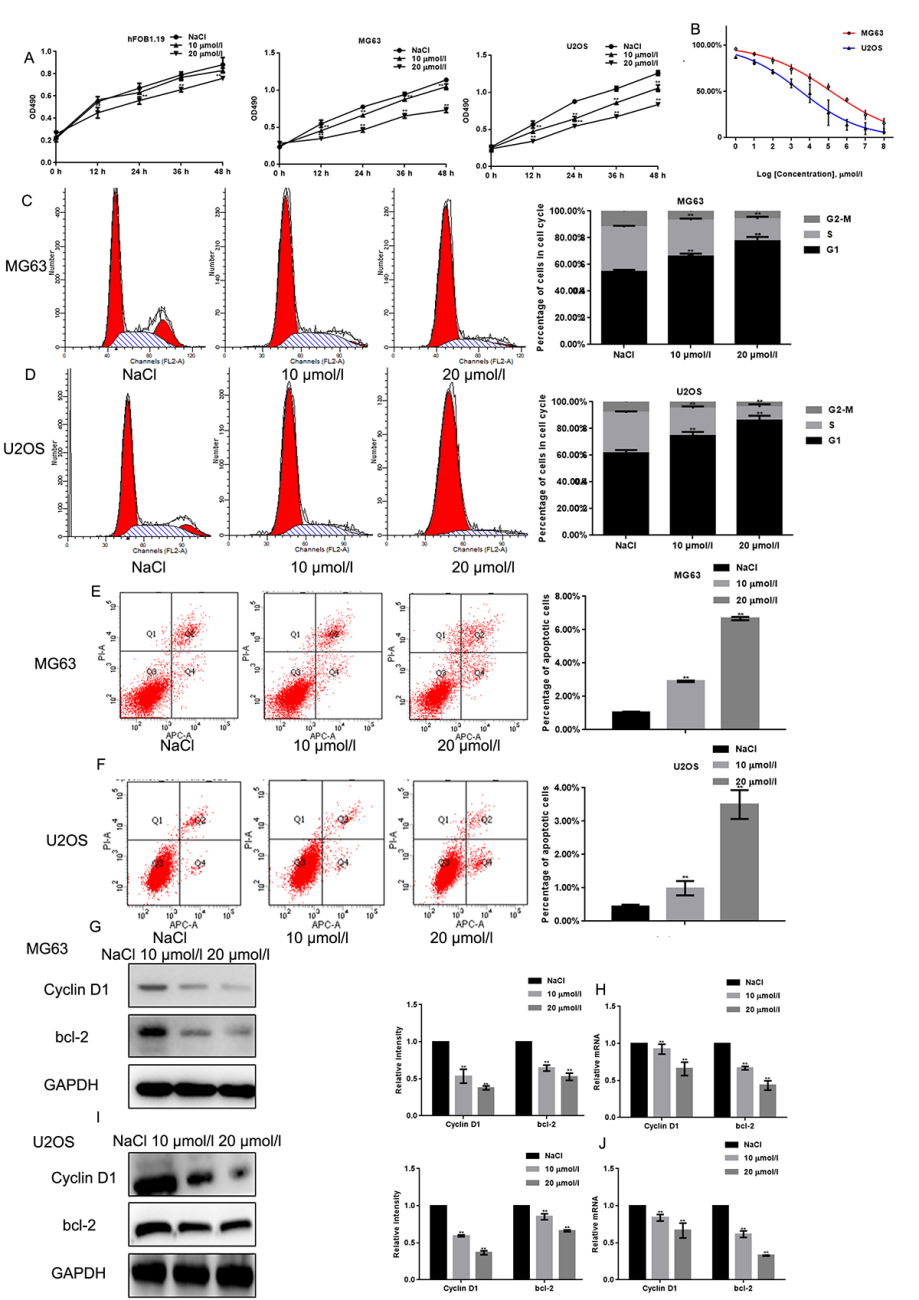
**Naringin inhibits the proliferation of osteosarcoma cells.** (**A**) Results of MTT proliferation assays in hFOB1.19, MG63, and U2OS cells cultured with various concentrations of naringin for different times. Results represent the mean ± SD of three experiments done in triplicate. **P < 0.05, compared with NaCl. (**B**) Proliferation inhibition rates induced by naringin on MG63 and U2OS cells. IC_50_ values were calculated through linear regression. (**C, D**) Flow cytometric analysis of cell cycle distribution in MG63 and U2OS cells pre-incubated with or without naringin for 24 h and stained with PI. The experiment was repeated three times. **P < 0.05, compared with NaCl. (**E, F**) Flow cytometric assay of apoptosis in MG63 and U2OS cells pre-incubated with or without naringin for 24 h and stained with Annexin V-FITC/PI. The experiment was repeated three times. **P < 0.05, compared with NaCl. (**G-J**) Expression of Cyclin D1 and bcl-2 detected by Western blot and real-time PCR in MG63 and U2OS cells treated with NaCl or naringin for 24 h. **P < 0.05, compared with NaCl.

The MTT assay revealed that naringin treatment inhibited the proliferation of MG63 and U2OS cells in a concentration dependent manner (Fig. 2A). The inhibitory effect of naringin on the proliferation of hFOB1.19 was only obvious when the concentration of naringin was 20 μmol/L. The IC50 of naringin on MG63 and U2OS cells at 24 h was ~50 μmol/L and ~30 μmol/L, respectively (Fig. 2B). Next, we used flow cytometry to evaluate cell cycle staging in PI-stained MG63 and U2OS cells previously exposed to various concentrations of naringin for 24 h. Naringin induced a dose-dependent increase in the percentage of cells in G_1_ phase, and decreased the number of cells in S phase, compared to control (Figs. 2C, D). To assess whether naringin can promote apoptosis, flow cytometry was used in Annexin-V-FITC-stained osteosarcoma cells. Results showed a dose-dependent increase in apoptotic cells treated with naringin (Figs. 2E, F). In line with these antiproliferative and pro-apoptotic effects, both Western blot and real-time PCR assays showed that exposure to 10 or 20 μmol/L naringin for 24 h dramatically decreased the expression of Cyclin D1 and bcl-2 (Figs. 2G-J).

### Naringin inhibits migration of osteosarcoma cells

**Figure 3:**
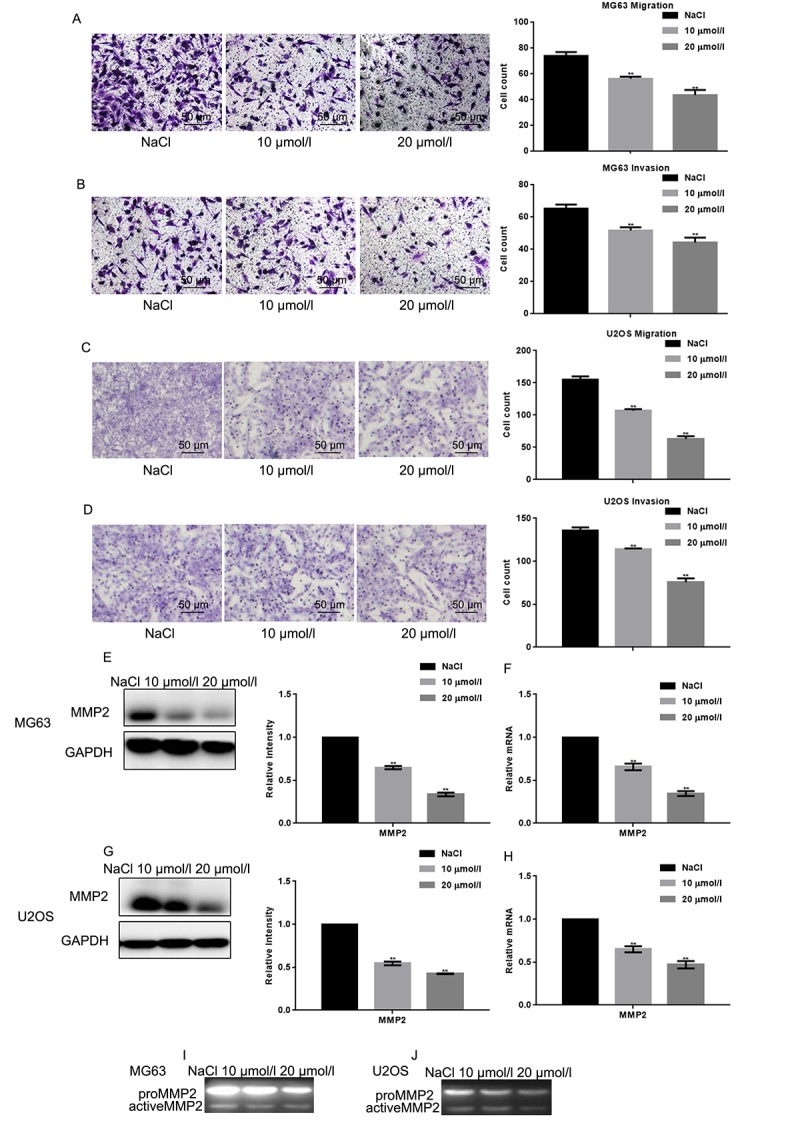
**Naringin inhibits migration and invasion of osteosarcoma cells.** (**A-D**) Migration and invasion were studied, respectively, using Transwell assays with or without Matrigel, in naringin-treated MG63 and U2OS cells. Cell count results represent the mean ± SD of three experiments. **P < 0.05, compared with NaCl. (**E-H**) MMP2 expression by Werstern blot and real-time PCR in MG63 and U2OS cells treated with NaCl or naringin for 24 h. **P < 0.05, compared with NaCl. (**I, J**) Zymography gel assay showing the inhibitory effect of naringin on MMP2 activity in MG63 and U2OS cells.

The effects of naringin on osteosarcoma cell migration and invasion was assessed using Transwell assays in the absence or presence, respectively, of Matrigel. Results showed that naringin exposure (10 or 20 μmol/L for 24 h) significantly decreased both migration and invasion of MG63 and U2OS cells in a dose-dependent manner (Figs. 3A-D). These effects were consistent with a decrease in MMP2 expression, detected in both cell lines in Western blot, real-time PCR, and zymography gel assays (Figs. 3E-H).

### Naringin suppresses osteosarcoma cell proliferation and migration by inhibiting Zeb1

**Figure 4:**
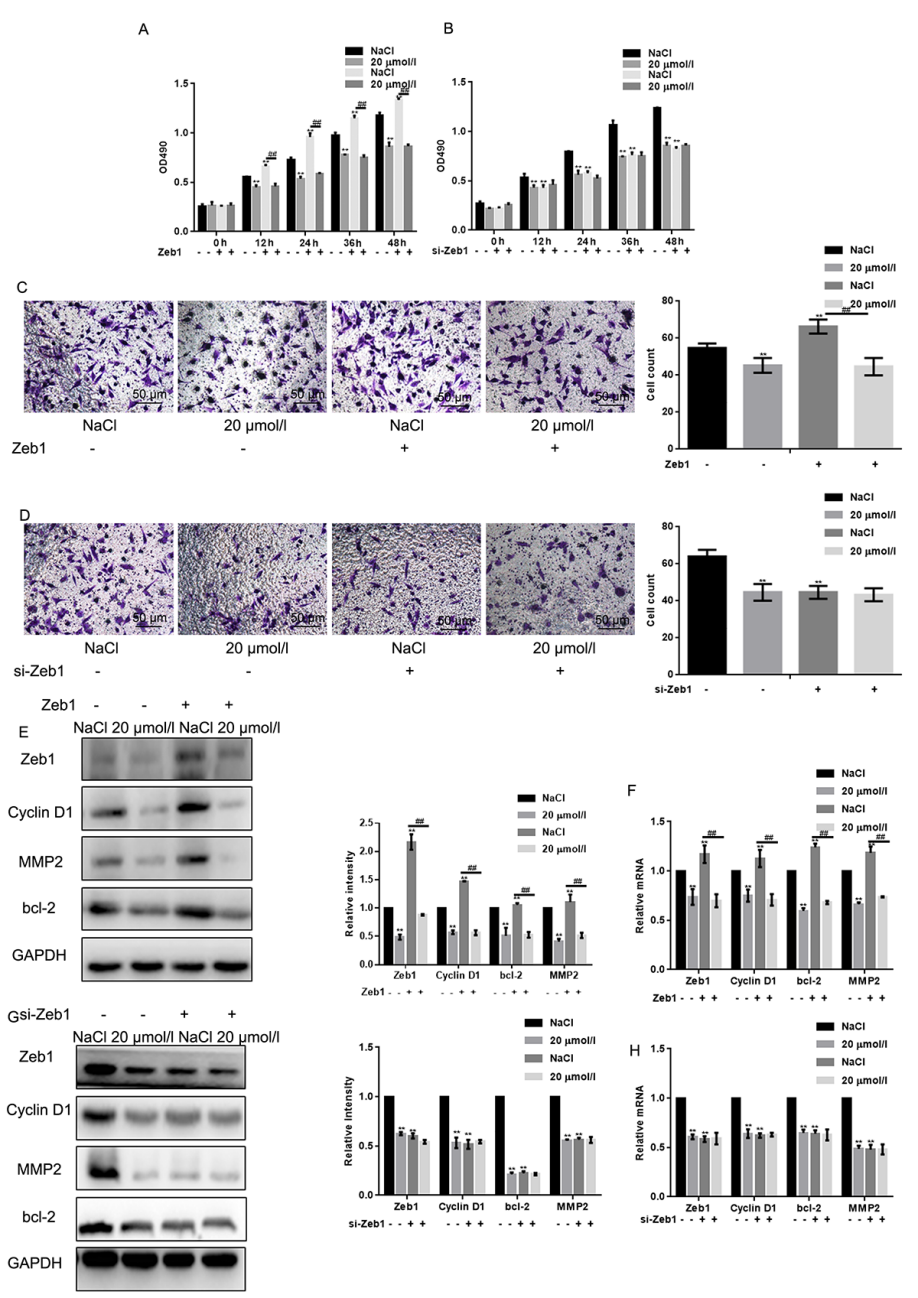
**Naringin suppresses osteosarcoma cell proliferation and migration by inhibiting Zeb1.** (**A**) MTT proliferation assay of MG63 cells expressing a control vector or Zeb1. Cells were incubated with 20 μmol/L of naringin or NaCl and assayed at the indicated times. Results represent the mean ± SD of three experiments done in triplicate. **P < 0.05, compared with NaCl. ## *P* < 0.05, compared with Zeb1 overexpressed group. (**B**) MTT proliferation assay of MG63 cells transfected with si-Zeb1 (Zeb1 silencing) or si-NC (negative control). Cells were treated with 20 μmol/L of naringin or NaCl and assayed at the indicated times. Results represent the mean ± SD of three experiments done in triplicate. **P < 0.05, compared with NaCl. (**C**) Results of Transwell migration assays (without Matrigel) performed in MG63 cells expressing control vectors or Zeb1. Cells were treated with 20 μmol/l of naringin or NaCl. **P < 0.05, compared with NaCl; ## *P* < 0.05, compared with Zeb1 overexpressed group. (**D**) Results of Transwell migration assays (without Matrigel) performed in MG63 cells transfected with si-Zeb1 (Zeb1 silencing) or si-NC (negative control). Cells were treated with 20 μmol/l of naringin or NaCl. **P < 0.05, compared with NaCl. (**E, F**) Western blot and real-time PCR assay results for Zeb1, Cyclin D1, bcl-2, and MMP2 expression in MG63 cells expressing Zeb1 or empty vector. Cells were incubated with 20 μmol/l of naringin or NaCl (control). **P < 0.05, compared with NaCl; ## *P* < 0.05, compared with Zeb1 overexpressed group. (**G, H**) Western blot and real-time PCR assay results for Zeb1, Cyclin D1, bcl-2, and MMP2 expression in MG63 cells transfected with si-Zeb1 or si-NC. Cells were treated with 20 μmol/l of naringin or NaCl. **P < 0.05, compared with NaCl.

To test the hypothesis that naringin exerts antiproliferative and anti-invasive effects by inhibiting Zeb1, its effects were tested in MG63 cells transfected with a plasmid encoding Zeb1 (Zeb1 overexpression) or an empty vector backbone (control). Conversely, siRNAs were introduced to downregulate Zeb1 (si-Zeb1), and to serve as non-targeted, negative control (si-NC). MTT assays showed that proliferation was stimulated by Zeb1 overexpression, and decreased to control inhibition levels by naringin (20 μmol/L) (Fig. 4A). On the other hand, Zeb1 suppression, although partial, decreased cell proliferation, and naringin induced no further inhibition in these cells. (Fig. 4B). We next examined whether migration was affected in Zeb1-overexpressing and Zeb1-suppressed cells. Transwell assay results indicated that Zeb1 overexpression enhances the migration of MG63 cells, while Zeb1 silencing recapitulates the inhibitory effect of naringin on control cells (Figs. 4C, D). Finally, we analyzed the effects of Zeb1 overexpression and downregulation on Cyclin D1, MMP2, and bcl-2 expression. The results showed that expression of these proteins increased upon Zeb1 upregulation (Figs. 4 E-F). Meanwhile, Zeb1 silencing lowered protein expression to levels like those observed in naringin-treated control cells (Figs. 4 G-H).

### Naringin inhibits the invasion of MG63 cells *in vivo*

**Figure 5:**
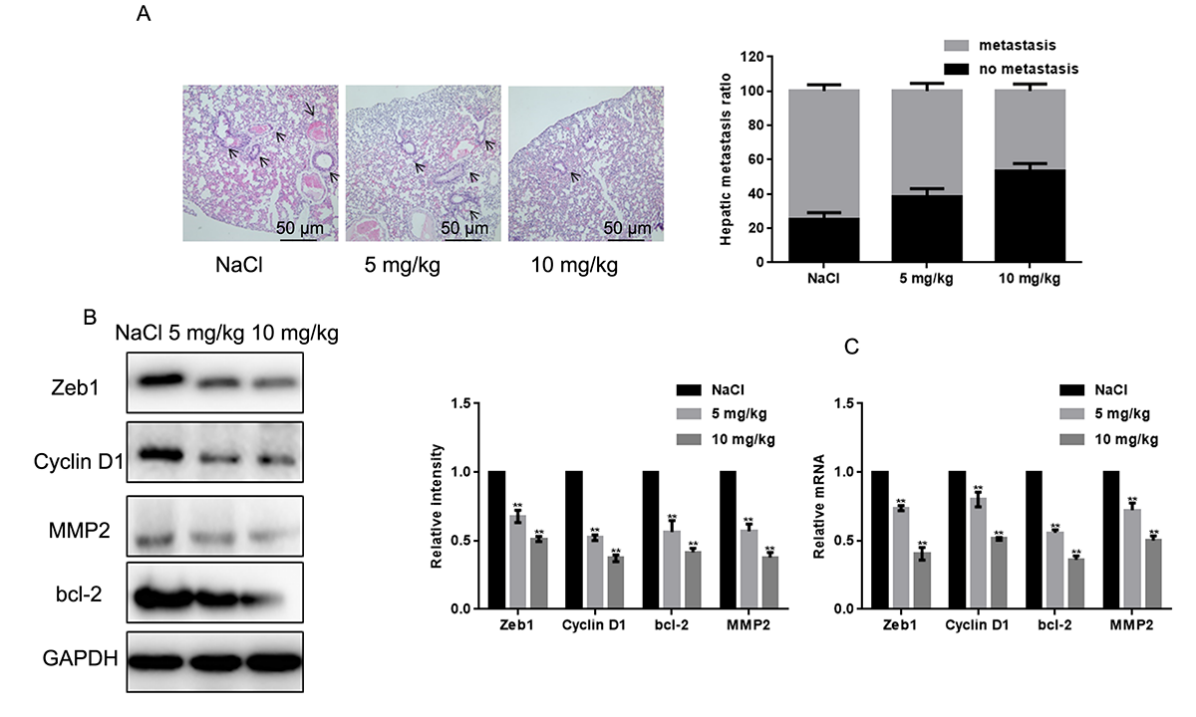
**Naringin inhibits lung invasion by MG63 cells *in vivo.*** (**A**) Representative images of lung histopathology (H&E staining; 400×) from mice injected with MG63 cells and treated daily with two different doses of naringin or NaCl (control) for 16 days. (**B, C**) Zeb1, Cyclin D1, bcl-2, and MMP2 expression in liver tumor samples, detected by Western blot (B) and real-time PCR (C). **P < 0.05, compared with NaCl-treated controls.

To examine the effect of naringin on osteosarcoma cell tumorigenesis *in vivo*, MG63 cells were injected into nude mice via the tail vein. After daily administration of naringin (5 or 10 mg/kg) or 0.9% NaCl for 16 days, mice were sacrificed and lung tissues processed for microscopic histological analysis. Results showed that naringin significantly prevented lung degeneration and reduced the incidence of metastatic nodules (Fig. 5A). Moreover, Western blots (Fig. 5B) and real-time PCR assays (Fig. 5C) showed that the expression of Zeb1, Cyclin D1, MMP2, and bcl-2 was decreased in the livers of mice treated with naringin.

## DISCUSSION

Naringin, a flavonoid present in citrus fruits, has HMG-CoA reductase inhibitor activity, and at low (nM) doses increases osteogenic activities in an osteoblast cell model *in vitro* [22]. Moreover, naringin-induced osteogenic differentiation has been recently described in bone marrow stromal cells and stem cells [23].On the other hand, naringin has been shown to inhibit cell proliferation and promote cell apoptosis in breast cancer, cervical cancer, melanoma, and bladder cancer cells [24]. Thus, we speculated that naringin may have therapeutic effect on osteosarcoma as well.

In this study, we tested the hypothesis that naringin has anticancer actions through inhibition of Zeb1, a zinc finger homeodomain transcription factor implicated in invasiveness and metastasis development in several tumor types, including osteosarcoma and lung cancer [25–27]. We first confirmed high Zeb1 expression in human specimens and in human MG63 and U2OS osteosarcoma cell lines. In these cells, naringin dose-dependently inhibited the expression of Zeb1, reduced proliferation by arresting the cell cycle in the G1 phase, and promoted apoptosis. In contrast, naringin had weaker effects on normal osteoblasts. The induction of apoptosis by naringin correlated with a decrease in anti-apoptotic bcl-2 protein expression; the latter was also observed in a study evaluating naringin’s effects in an ovarian cancer mouse model [12]. Naringin-mediated apoptosis has been documented in cervical cancer cells, in two studies that implicated NF-κB/COX-2-caspase-1 pathway repression [9] and expression of caspases, p53, Bax, and Fas death receptor [28] respectively.

Our results also showed that both naringin exposure and Zeb1 silencing significantly suppressed osteosarcoma cell migration in Transwell assays. This is consistent with the down-regulation of MMP2, observed under both conditions, and the stimulation of MMP2 expression seen instead after ectopic expression of Zeb1 in these cells. These effects may be related to a well-known role of Zeb1 in promoting metastasis through epithelial-to-mesenchymal transition (EMT), although more research is needed to clarify the mechanisms at play in osteosarcoma [29].

Importantly, we demonstrated that naringin’s effects *in vitro* correlated with antimetastatic actions *in vivo*, as its administration to nude mice injected with osteosarcoma MG63 cells attenuated the formation of tumor nodules in the liver. In summary, our data showed that naringin inhibits the malignant phenotype of osteosarcoma cells by inhibiting the expression of Zeb1 and Zeb1-associated proteins such as Cyclin D1 and MMP2. The present findings support the potential of naringin as a novel therapeutic strategy for osteosarcoma.

## MATERIALS AND METHODS

### Drugs and reagents

Naringin, 3-(4,5-dimethyl-2-thiazolyl)-2,5-diphenyl-2-H-tetrazolium bromide (MTT), propidium iodide (PI), and Trypan Blue were purchased from Beyotime Biotechnology (Shanghai, China). Fetal bovine serum (FBS), Dulbecco’s modified Eagle’s medium (DMEM), Lipofectamine 2000, and TRIzol were obtained from Invitrogen (Carlsbad, CA, USA). RT-PCR kits were obtained from Promega (Beijing, China). SYBR Premix Ex Taq reagents were obtained from TaKaRa (Dalia, China). Anti-Zeb1, anti-cyclin D1, anti-bcl-2, anti-MMP2, and anti-GAPDH antibodies were purchased from Santa Cruz Biotechnology (Dallas, TX, USA). HRP-conjugated secondary antibody, BCA protein assay kit, and enhanced chemiluminescence (ECL) solution were purchased from Beyotime Biotechnology. All experiments were completed in the Central Laboratory of our hospital.

### Human samples

Tissue samples were obtained from patients undergoing surgery at Shengjing Hospital of China Medical University (Table 1). The original histopathologic reports were obtained from each case, and the diagnosis of osteosarcoma was confirmed. Part of the excised tissue was embedded in paraffin, and part of the sample was snap-frozen at −80°C. Clinical samples were collected after written informed consent was obtained, and the study was approved by the Ethics Committee at the Academic Medical Center of Shengjing Hospital of China Medical University.

**Table 1:** Patient information.

**Group**		**N**	**Percent**
Sex	Male	13	65%
	Female	7	35%
Age	<20	12	60%
	>20	8	40%
History	Yes	1	5%
	No	19	95%
Site of primary disease	Tibia	11	55%
	Femur	7	35%
	Humerus	2	10%
TNM	I	9	45%
	II	7	35%
	III	5	25%

### Cell culture

Human osteosarcoma MG63 and U2OS cells, and human hFOB1.19 osteoblasts (SV40-transfected), used as control, were supplied by the Cell Pool Bank of China (Guangzhou, China). The cells were cultured in DMEM supplemented with 10% FBS at 37°C under an atmosphere of 5% CO_2_ and 95% air.

### Cell viability assay

The MTT assay was employed to assess cell viability. Cells were cultured in 96-well plates at a concentration of 1×10^4^ cells/ml and incubated with 10 or 20 µmol/L naringin for 12, 24, 36, or 48h. At those time intervals, 0.01 ml of MTT solution (5 mg/ml) was added to each well. After a 4 h incubation at 37 °C, medium was replaced by 0.15 ml DMSO. After 15 min incubation at 37 °C, optical densities (490 nm) were measured.

### Cell cycle assay

Cells were incubated with 10 or 20 µmol/L naringin for 24 h and fixed in 75% ethanol at 4°C overnight. After resuspension in 10 μg/ml PI, cell cycle stages were determined using a FACS Vantage ﬂow cytometer using CellQuest (Becton Dickinson and Co., San Jose, CA, USA).

### Apoptosis assay

Cells were incubated with 10 or 20 µmol/L naringin for 24 h, washed twice with cold PBS, and stained with 5 μl ANNEXIN-V-FITC/10 μl PI for 15 min. After addition of 400 μl binding buffer to each tube, the apoptosis rate was measured by flow cytometry within 1 h.

### Transwell migration assay

Transwell assays were performed using a modiﬁed Boyden chamber with Nuclepore polycarbonate membranes. After 24 h treatment with 10 or 20 µmol/L naringin, 1×10^5^ cells in 100 μl FBS-free DMEM were placed in the upper part of the chamber with or without Matrigel, whereas the lower compartment was ﬁlled with 600 μl DMEM containing 10% FBS. After 8 h incubation at 37°C, the invading cells on the lower surface of the ﬁlter were ﬁxed, stained with Trypan Blue, and counted under high-power magniﬁcation (400×).

### Zymography

Cells were cultured in 12-well plates and treated with 10 or 20 µmol/L naringin. After 24 h, media was changed into DMEM containing 5% FBS (the source of proMMP2). After another 24 h, the media were harvested, cleared by centrifugation at 12,000 rpm for 10 min, and subjected to analysis by SDS-PAGE impregnated with 1 mg/ml gelatin. The gels were incubated at 37°C overnight, stained with Coomassie Blue, destained, and then scanned.

### Transfection

To stably overexpress and silence Zeb1, cells were transfected with a pcDNA3.1 vector encoding Zeb1, and with a Zeb1-targeted siRNA, respectively (Shanghai GeneChem Company, Shanghai, China). An empty pcDNA3.1 vector and a non-targeted siRNA were transfected as respective controls, and cells were selected with puromycin (1.5 μg/mL). Lipofectamine 2000 was used for cell transfection according to the manufacturer's protocols. We extracted protein and total RNA at 24 h after transfection.

### Real-time PCR

Total RNA was extracted after the indicated treatments (24 h) using TRIzol according to the manufacturer’s protocol. Cells or tissues were lysed by 0.2 mL chloroform and centrifuged (12,000 × g at 4°C for 15 min). The supernatant was then treated with 0.5mL isopropanol and centrifuged (12,000 × g at 4°C for 10 min). The RNA pellet was dissolved in 1 mL 75% ethanol, centrifuged (7,500 × g at 4°C for 5 min), and the supernatant discarded. After resuspension in DEPC water, 1 µg of RNA was reverse transcribed to cDNA using a RT-PCR kit. Real-time PCR was performed using an Mx 3000P real-time PCR system (Applied Biosystems, Shanghai, China) and SYBR Premix Ex Taq as a DNA-speciﬁc ﬂuorescent dye. PCR was carried out for 40 cycles of 95°C for 10 s and 60°C for 30 s. Primer sequences for detection of mRNA expression were synthesized (Table 2). All the reactions were repeated at least three times. Gene expression levels were calculated relative to the housekeeping gene GAPDH using Stratagene Mx 3000P software.

**Table 2:** Primers for RT-PCR.

**Name**	**Forward primer (5'->3')**	**Reverse primer (5'->3')**
Zeb1 (NM_001323643.1)	GCACAACCAAGTGCAGAAG	CATTTGCAGATTGAGGCTG
Cyclin D1 (NM_053056.2)	CCGAGGAGCTGCTGCAAATGGAGCT	TGAAATCGTGCGGGGTCATTGCGGC
MMP2 (NM_004530.5)	CGCATCTGGGGCTTTAAACAT	TCAGCACAAACAGGTTGCAG
GAPDH (NM_002046.6)	GAAGGCTGGGGCTCATTTG	AGGGGCCATCCACAGTCTTC

### Western blot

Tissues (homogenized by grinding) and treated cells were lysed with lysis solution at 4°C for 30 min, followed by centrifugation (12,000 × g at 4°C for 15 min). From each sample, 20 µg of protein was fractionated by 10% sodium dodecyl sulphate-polyacrylamide gel electrophoresis (SDS-PAGE) and transferred onto polyvinylidene difluoride (PVDF) membranes (Amersham, Beijing, China). After blocking with 5% nonfat dry milk in TBST for 1 h at room temperature, proteins were probed with specific antibodies against Zeb1, Cyclin D1, or MMP2. To assure equal loading, gels were stripped and reprobed with an anti-GAPDH antibody. Following incubation with HRP-conjugated secondary antibodies, signals were detected by chemiluminescence. All the reactions were repeated at least three times.

### *In vivo* experiments

Five- to six-week-old female, athymic nude BALB/c mice (Vital River Laboratory Animal Technology Co. Ltd., Shanghai, China) were split into three groups of six and received tail vein injections containing 2×10^6^ MG63 cells in 0.1 ml saline. The following day, and once a day thereafter, the mice were given intravenous injections of naringin (5 or 10 mg/kg) or 0.9% NaCl (control). On day 16 following tumor cell injection, liver samples were collected for histological examination.

All experimental procedures involving animals were conducted in accordance with the Guide for the Care and Use of Laboratory Animals (NIH publication no. 80-23, revised 1996) and followed institutional ethical guidelines. The study was approved by the Ethics Committee at the Academic Medical Center of Shengjing Hospital of China Medical University.

### Histopathology

Lung specimens were fixed with 4% paraformaldehyde. Serial sections were cut using a microtome and affixed onto positively charged slides. Tissues were deparaffinized and rehydrated through graded xylene and alcohol. The sections were lightly counterstained with hematoxylin–eosin, dehydrated through an ethanol series, cleared in xylene and mounted. Stained sections were viewed using a light microscope (400×).

### Statistical analysis

All data are presented as the mean ± SD. Statistical significance between two groups of data was evaluated by Student's t test (two-tailed) using GraphPad Prism software (GraphPad, Inc., La Jolla, CA, USA). *P* < 0.05 was considered significant.

### Ethics statement and consent to participate

Research involving human subjects, human material, or human data has been performed in accordance with the Declaration of Helsinki and was approved by the Research Ethics Committee of Shengjing Hospital (R20160965).

### Compliance with ethical standards

For the use of clinical materials for research purposes, written consent and approval from patients were obtained from the Shengjing Hospital of China Medical University. Patient consent was obtained in writing according to institutional regulations.

### Consent to publish

We have obtained consent to publish from the participants to report individual patient data.
